# Efficacy of Novel Digital-Based Surgical Guide in the Limited Interocclusal Distance

**DOI:** 10.3390/bioengineering11121177

**Published:** 2024-11-21

**Authors:** Won-Jong Park, Ki-Seong Kim, Seok-Hwan Cho, Su Young Lee

**Affiliations:** 1 Department of Oral and Maxillofacial Surgery, Seoul St. Mary’s Hospital, College of Medicine, The Catholic University of Korea, Seoul 06591, Republic of Korea; roll8888@naver.com; 2 NamSang Dental Clinic, 118 Shinpung-ro, Yeongdeungpo-gu, Seoul 07432, Republic of Korea; namsang0249@nate.com; 3 Department of Prosthodontics, The University of Iowa College of Dentistry and Dental Clinics, 801 Newton Rd., Iowa City, IA 52242, USA; seokhwan-cho@uiowa.edu; 4 Department of Prosthodontics, Seoul St. Mary’s Hospital, College of Medicine, The Catholic University of Korea, Seoul 06591, Republic of Korea

**Keywords:** dental implants, accuracy, computer-aided design (CAD), surgical guide, printing

## Abstract

Accurate implant placement is essential for achieving successful outcomes. To aid in this, digitally designed surgical guides have been introduced. Both closed-sleeve and open-sleeve designs are commonly utilized. However, the closed-sleeve design has limitations with restricted interocclusal distance, interference with irrigation, and limited visibility, while the open-sleeve design is known to be less accurate. To address these limitations, a new slope-sleeve design was introduced. This design reduces the interocclusal distance requirement compared to the closed-sleeve design and provides improved accuracy. A constraint model with a 31 mm interocclusal distance was created, and three types of surgical guides (closed, open, and slope), printed using either a PolyJet or Digital Light Processing (DLP) 3D printer, were tested on resin bone blocks. Horizontal and angular deviations were measured for the accuracy of each guide after drilling, with data analyzed using one-way ANOVA and independent *t*-tests. The slope-sleeve design showed significantly lower horizontal and angular deviations in wide-sized guides. Additionally, PolyJet-printed guides showed higher accuracy compared to DLP-printed guides. The slope-sleeve guide offers enhanced stability and precision in restricted interarch spaces. When coupled with high-precision 3D printing technologies like PolyJet, the slope-sleeve design provides a reliable solution for improving implant placement accuracy in challenging clinical scenarios.

## 1. Introduction

Placing implants in precisely planned positions is essential for the fabrication of prosthetics to achieve optimal aesthetic and functional restoration for patients [1,2,3]. Various methods have been attempted for this purpose over the years. Recently, advancements in digital technology have enabled digitization of prosthetic information, allowing for the planning of implant positions in a virtual environment using CT and model scan data. Furthermore, it is now possible to pre-position the final prosthetics in the virtual space and develop an implant placement plan that takes these into account [4,5]. The tool that allows installation of implants in a patient’s oral cavity according to planned positions is called the surgical guide [6,7]. In the past, these guides were manually crafted based on the traditional plaster model, but with the advent of digital technology, the production of guides using digital CAD/CAM has become predominant [5,7,8,9,10]. These advancements have significantly enhanced the accuracy and efficiency of implant placements, reducing the potential for errors and improving patient outcomes [4,11,12,13,14,15].

Despite these improvements, there are limitations in applying digital surgical guides to all patients. Typically, surgical guides use metal sleeves, which offer advantages in terms of accuracy. However, they can obstruct irrigation during surgery, leading to bone heating. They do not allow direct visualization of the drilling site [16]. Additionally, the drill must be inserted vertically into the metal sleeve, which increases the interocclusal distance required during the procedure [17]. Specifically, in the limited interach space, insufficient mouth opening restricts the use of surgical guides. Depending on the implant system, there is a minimum height required to use the guide. If the mouth opening in the posterior region does not meet this minimum distance, the guide becomes impractical to use [17,18,19,20].

To address these issues, an open-sleeve-type surgical guide has been developed. Unlike the traditional closed-sleeve guide, this guide features an opening on the buccal or opposite side. This design allows the drill to enter laterally into the sleeve, rather than only accessing the top of the sleeve parallel to the implant placement direction. Additionally, the opening enables direct visualization of bone inside the guide, improving surgical field of view and enhancing irrigation efficiency [17,21].

Although the open-sleeve-type surgical guide allows lateral movement, it has accuracy issues. This is because, during drilling, the instrument may lean toward the open side, and, particularly in the posterior region, if the head of the handpiece contacts the opposing teeth, it may tilt buccally from the planned angle. Previous studies have reported a decrease in accuracy when using open-type sleeves in the posterior region. However, there are also reports indicating that the open-type can provide high accuracy by allowing surgeons better drill control, particularly depending on the shape of the extraction socket [21,22].

In this study, we proposed a new design of surgical guide, the slope-sleeve type, aiming at reducing the minimum interocclusal distance requirement and increasing stability. Limited interocclusal distance refers to the restricted vertical space between the maxilla and mandible when the mouth is opened. This limitation, commonly found in posterior regions, can impede the accessibility and positioning of surgical tools. This new guide is expected to enhance drill accessibility in narrow spaces due to interocclusal distance. The sloped hole structure is anticipated to increase accuracy by providing additional lateral support during drilling.

The purpose of this study was to validate the accuracy to the osteotomy process of the new slope-sleeve type compared to conventional open- and closed-sleeve-type surgical guides, particularly in situations with limited interocclusal distance. The second goal was to evaluate the accuracy of surgical guides based on the fabrication method of the 3D printer used.

## 2. Materials and Methods

### 2.1. Design of Interocclusal Distance Constraint Model

**Figure 1:**
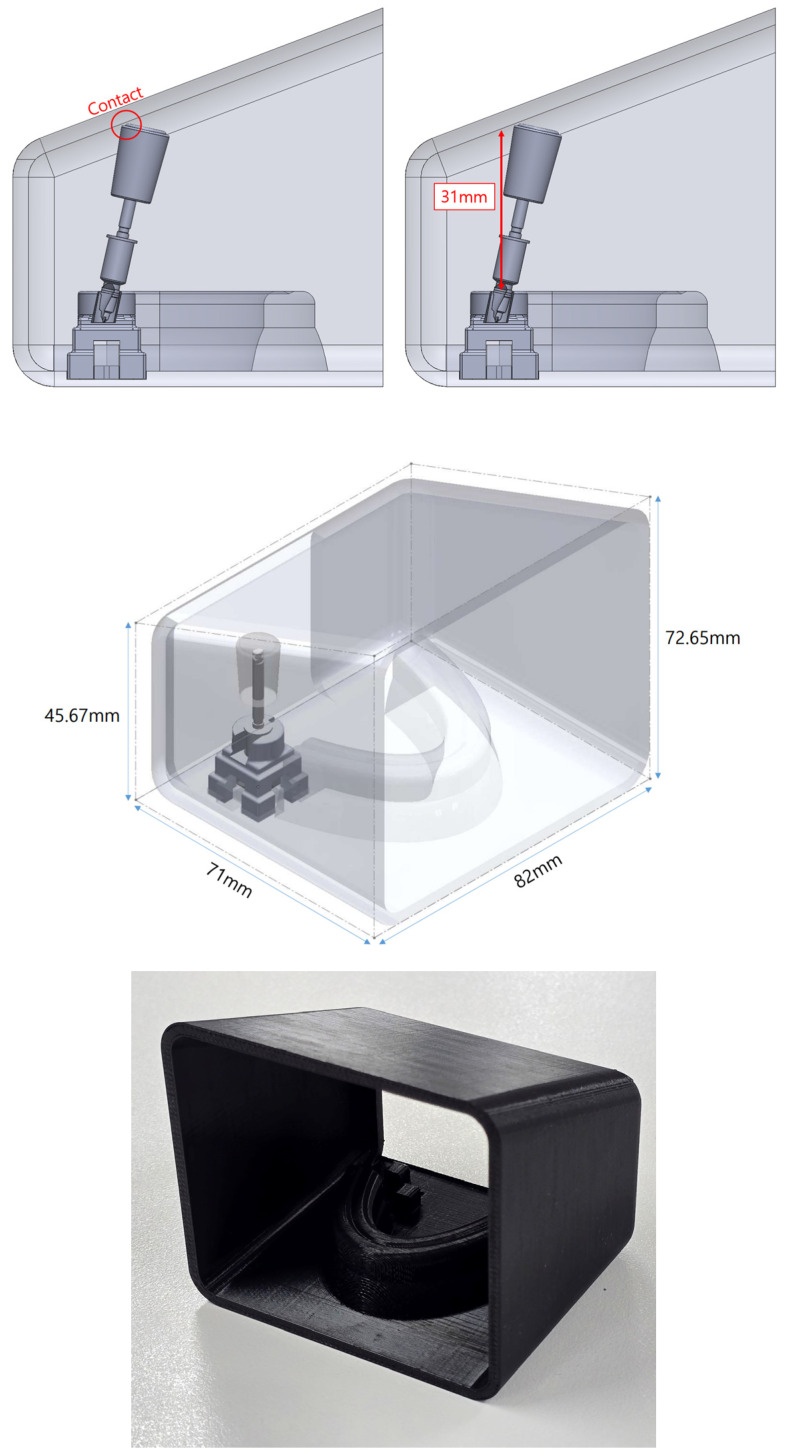
Design of interocclusal distance constraint model.

To impose restrictions on the entry of the drill into the surgical guide, the rectangular three-dimensional structure incorporating the arch was created based on the size of the virtual oral model. In this structure, the interocclusal distance was limited to 31 mm. The structure was fabricated with a 3D printer (F120, Stratasys, MN, USA) using ABS Black material (Figure 1).

### 2.2. Specimen Prepartion

**Figure 2:**
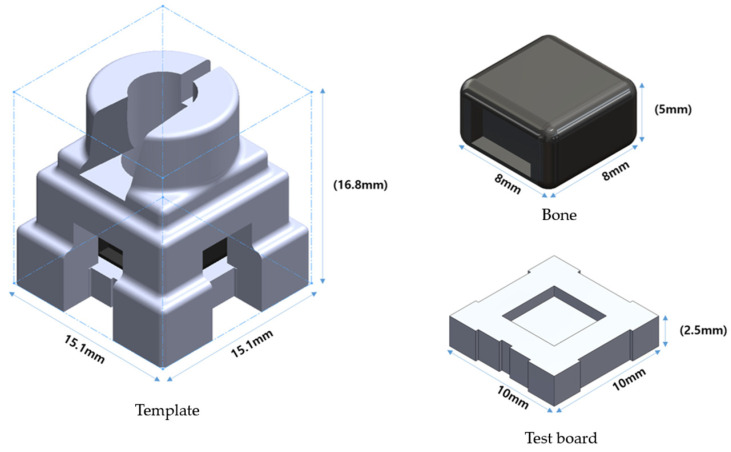
Template, bone, and test board design.

To measure angular and horizontal deviation, a specimen was fabricated consisting of a surgical guide template, a resin bone block, and a test board (Figure 2).

A surgical template component serves as a guide for drill entry and direction. Three types of guide templates (closed-hole, open-hole, and slope-hole) were printed, each in both regular and wide sizes (*n* = 10). To verify the differences in accuracy according to the production method, these were produced using both the PolyJet method (Eden350V, Stratasys, MN, USA) and Digital Light Processing (DLP) (Onejet DLP Plus, Osstem implant, Seoul, Republic of Korea).

The idea behind the slope guide is not only to create space by angling the drill anteroposteriorly but also to take advantage of the hinge structure of the oral cavity, where the interocclusal distance increases toward the anterior as the mouth opens. This design leverages both the space created by tilting and the additional room gained from positioning the handpiece head further forward, allowing more space for instruments.

**Figure 3:**
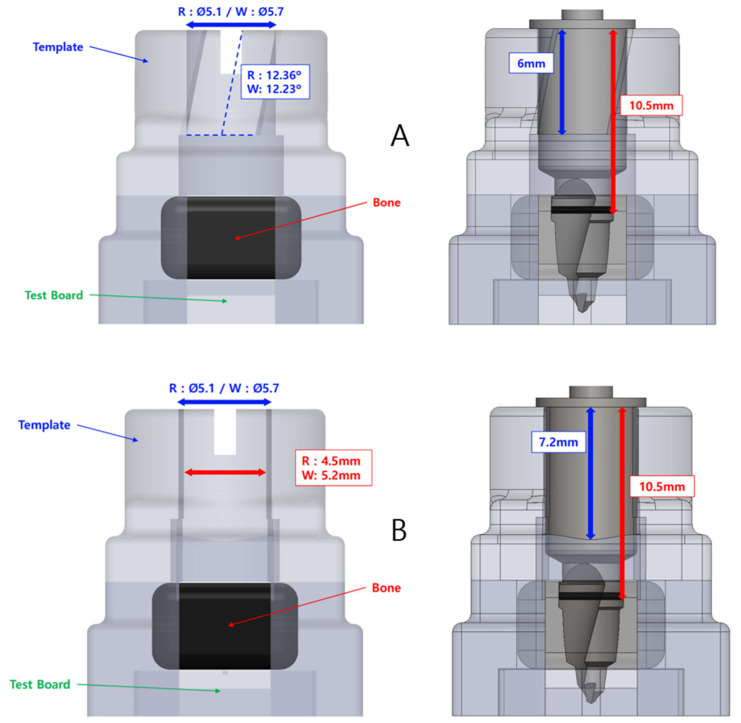
Experimental model design. (**A**) Slope type; (**B**) open type.

The slope guide has an internal height of 6.0 mm and is designed to be 10.5 mm from the top of the bone part. The hole has an internal diameter of Ø5.1 mm for regular and Ø5.7 mm for wide. The inclination of the slope type was specified as 12.36 degrees and 12.23 degrees, respectively, with a maximum value that does not exceed the hole diameter (Figure 3).

A resin bone block was fabricated with 3D printing resin (ABS Black, Stratasys, MN, USA) using the fused deposition modeling (FDM) method. The block, sized 8 × 8 × 5 mm, features a lattice structure with an outer (cortical) thickness of 0.762 mm and an inner (cancellous) thickness of 0.254 mm simulated type 2 bone.

A test board of 10 × 10 × 2.5 mm was printed using a PolyJet (Eden350, Stratasys, MN, USA) to allow the drill tip to penetrate to a depth of 1 mm.

**Figure 4:**
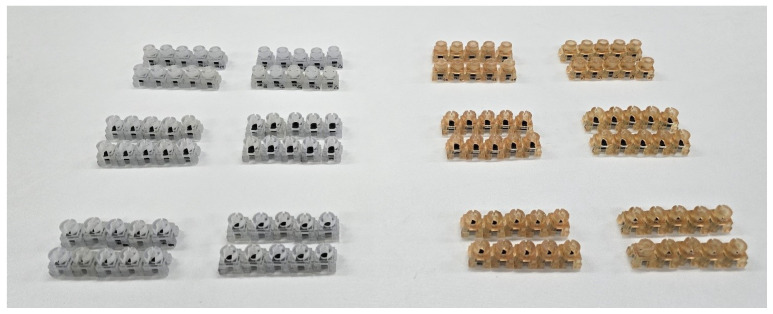
Experimental specimens after assembly following 3D printing (left: PolyJet; right: Digital Light Processing).

**Figure 5:**
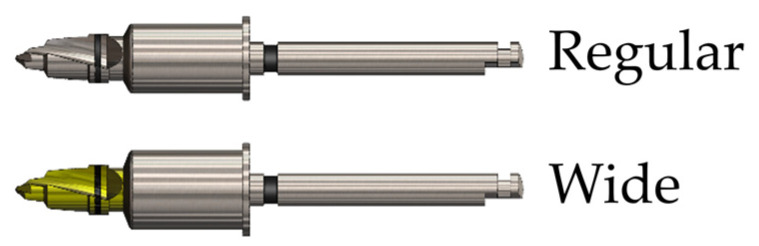
OneGuide Initial Drill (Osstem Implant, Seoul, Republic of Korea).

The resin bone block and test board components were used consistently across experiments, while the surgical template was varied in the design. A total of 120 sets of specimens were produced (Figure 4). For the osteotomy procedure, an initial guide drill (OneGuide, Osstem, Seoul, Republic of Korea) was utilized. The guide drill entered the surgical guide and drilled through the resin bone block in 5 mm and test board in 1 mm. The shape of the drill is illustrated in Figure 5, featuring a hole diameter of 5.1 mm for a regular and 5.7 mm for a wide drill. The drill barrel and guide sleeve have a drill tolerance of 0.05 mm per side, totaling 1 mm, which allows for irrigation during surgery and prevents binding of the drill, thereby reducing frictional heat generation.

### 2.3. Verification Method

The specimen was mounted on the most posterior area of an interocclusal distance constraint model. Drilling was performed with the initial drill (OneGuide, Osstem, Seoul, Republic of Korea) at 800 rpm using the dental handpiece (W&H Implantmed, Bürmoos, Austria). After osteotomy procedures, the specimen was disassembled to obtain resin bone and test board components.

The bone component was used to measure angular errors. A pin gauge was inserted into the hole formed, and the maximum inclination was measured from four directions using a profile projector (HB400, Starrett, Athol, MA, USA).

**Figure 6:**
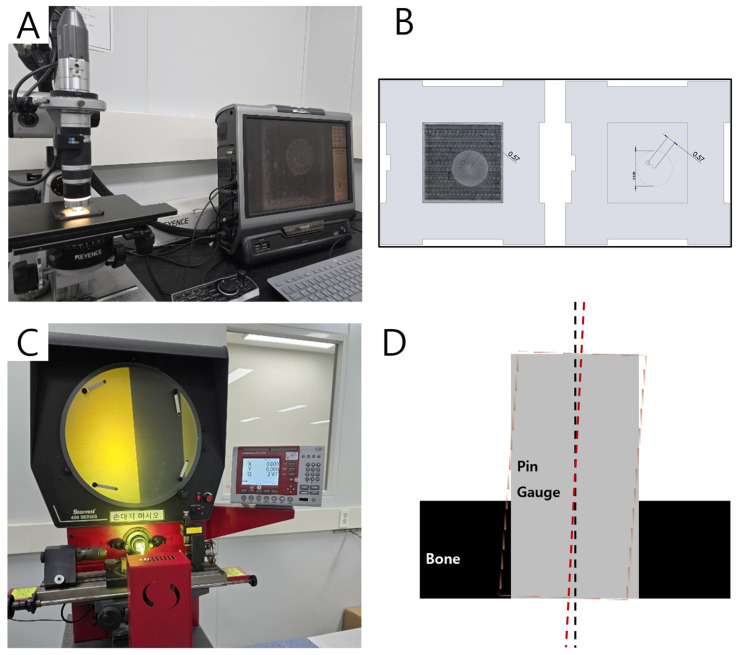
(**A**) The microscope used for measuring horizontal error; (**B**) the method of measuring horizontal error; (**C**) the projector used for measuring angular error; (**D**) the method of measuring angular error.

The test board was used to measure horizontal errors. The drill marks on the test board were photographed under an electron microscope (VHX-1000, KEYENCE, Osaka, Japan). Images were uploaded into CAD software (Solidworks, https://www.solidworks.com/, Dassault Systèmes, Vélizy-Villacoublay, France) to measure displacement of the drilling center relative to the origin (Figure 6).

### 2.4. Statistical Analysis

A total of 12 groups were analyzed, with each group containing 10 samples. Results are presented as mean ± standard deviation (SD). All statistical analyses were performed using SPSS software version 29 (IBM Corp., Armonk, NY, USA). Normality of data was evaluated with the Shapiro–Wilk test. Means for angular and horizontal distance differences for the three surgical guide designs (open-, closed-, or slope-type) were compared using a one-way ANOVA. Bonferroni correction was applied to adjust for multiple comparisons in post hoc tests. Independent *t*-tests (*n* = 60) were used to compare the means of the angular and horizontal distance differences of the surgical guides according to the 3D printing manufacturing method (PolyJet or DLP). All *p*-values were two-sided, with a significance threshold set at α = 0.05.

## 3. Results

### 3.1. Influence of Template Design on Accuracy of Drilling

**Figure 7:**
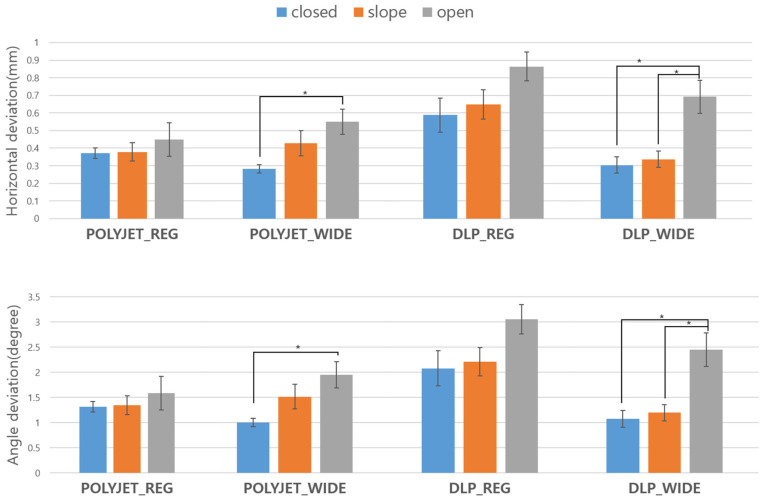
Graph of horizontal deviation and angle deviation of implant drilling with different surgical guides. * Significant at *p* < 0.05.

Horizontal deviation and angle deviation for each group are summarized in Table 1, Table 2, and Figure 7, respectively.

In the PolyJet wide group, the horizontal deviation showed significant differences between the closed type and the open type (*p* = 0.015). Similarly, in the DLP wide group, significant differences were observed between the closed type and the open type (*p* < 0.001), as well as between the slope type and the open type (*p* < 0.001). No statistically significant differences in horizontal deviation were found between PolyJet regular and DLP regular groups.

Angle deviation results mirrored those of the horizontal deviation. In the PolyJet wide group, significant differences were again noted between the closed type and the open type (*p* = 0.014). In the DLP wide group, comparisons between the closed type versus open type (*p* < 0.001) and between the slope type and the open type (*p* < 0.001) also showed statistically significant differences in angle deviation. However, the PolyJet regular and DLP regular groups did not exhibit any significant differences in angle deviation.

These findings indicate that, particularly in the wide configurations of both PolyJet and DLP groups, the open type significantly deviates in both horizontal and angular measurements compared to the closed type and slope type.

### 3.2. Accuracy of Surgical Guides According to 3D Printer (PolyJet or DLP)

**Figure 8:**
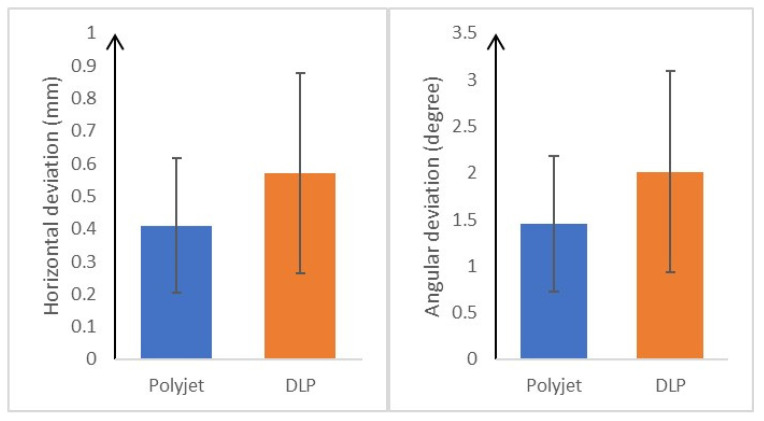
Graph of horizontal and angular deviation of surgical guides fabricated from PolyJet or DLP (mm or degree, mean ± SD). SD, standard deviation; DLP, Digital Light Processing. Significant at *p* < 0.05.

Surgical guides produced by the PolyJet 3D printer had statistically significantly lower deviations in both horizontal deviation (*p* < 0.001) and angular deviation (*p* = 0.001) than those produced by the DLP method (Table 3 and Figure 8).

## 4. Discussion

In this study, the accuracy of drilling for implant placement in the limited interdental arch using a surgical guide was investigated, focusing on the effect of sleeve design using an experimental model. Additionally, the study examined differences between different types of 3D printers. The results indicated that while no statistical significance was observed when using the regular diameter, the PolyJet group with the wide diameter showed a higher accuracy in the closed type than in the open type. In the DLP group, both the closed type and the slope type demonstrated higher accuracy than the open type. Across all groups, the open type tended to have the lowest accuracy. However, there were no significant differences in accuracy between the closed type and slope type.

Kholy et al. [23] have reported the accuracy of the closed type with deviations ranging from 0.713 mm to 1.225 mm at the crest, and from 0.891 mm to 1.352 mm at the apex, with angular deviations ranging from 2.747° to 4.319°. Similarly, Guentsch et al. [24] have reported accuracy of the closed type with deviations of 0.54 ± 0.28 mm at the crest, 0.67 ± 0.40 mm at the apex, and an angular deviation of 1.95 ± 1.51°. Although our study did not involve the placement of implants and the position of the apex after drilling and the drilling angle, the horizontal deviation in this study was smaller, while the angular deviation was similar to those reported in previous studies. This might be attributed to the fact that the values were measured after a single drilling with a short initial drill, resulting in smaller deviations.

Several studies have compared the accuracy of open-type and closed-type surgical guides. Oh et al. [25] have compared the accuracy in healed sockets based on the presence of a metal sleeve, using both open-type and closed-type surgical stents. They found that the buccolingual angular deviation was higher in the open type than in the closed type. Tallarico et al. [22] have performed a clinical trial on open-type surgical stents. They reported a lower accuracy with the open type, although they did not perform statistical analysis by type. Li et al. [21] have conducted experiments using two bone models: healed sockets and fresh sockets. They reported that the closed type had higher accuracy in healed sockets, whereas the open type showed better accuracy in fresh sockets. Chen et al. [23] have reported similar findings, showing that when the septum in the extraction socket was sufficiently present to encircle the implant, the closed type demonstrated higher accuracy. In contrast, when the septum was thinner, the open type provided better accuracy than the closed type due to its greater flexibility and enhanced visibility. When synthesizing results of previous studies, it is evident that the accuracy of the open type is lower in healed extraction sockets or models. Our experimental results aligned with this finding. Therefore, the new slope guide might be able to achieve higher accuracy in healed sockets as well as less-healed sockets or immediate placement due to better visibility and stability.

**Figure 9:**
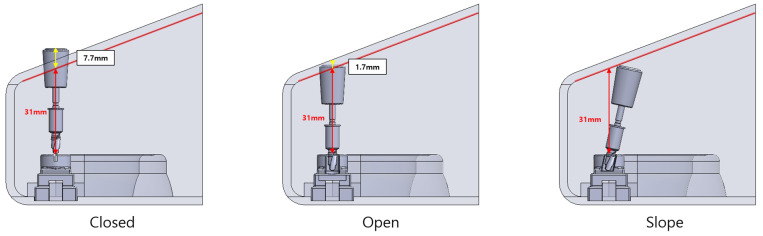
Comparison of minimum interocclusal distance by template type.

In addition, the newly designed slope-type surgical guide requires a shorter interocclusal distance than the conventional closed type and open type. This was achieved by applying an open-sleeve design angled toward the anterior region, leveraging the naturally increasing interocclusal distance as the mouth opened (Figure 9).

**Figure 10:**
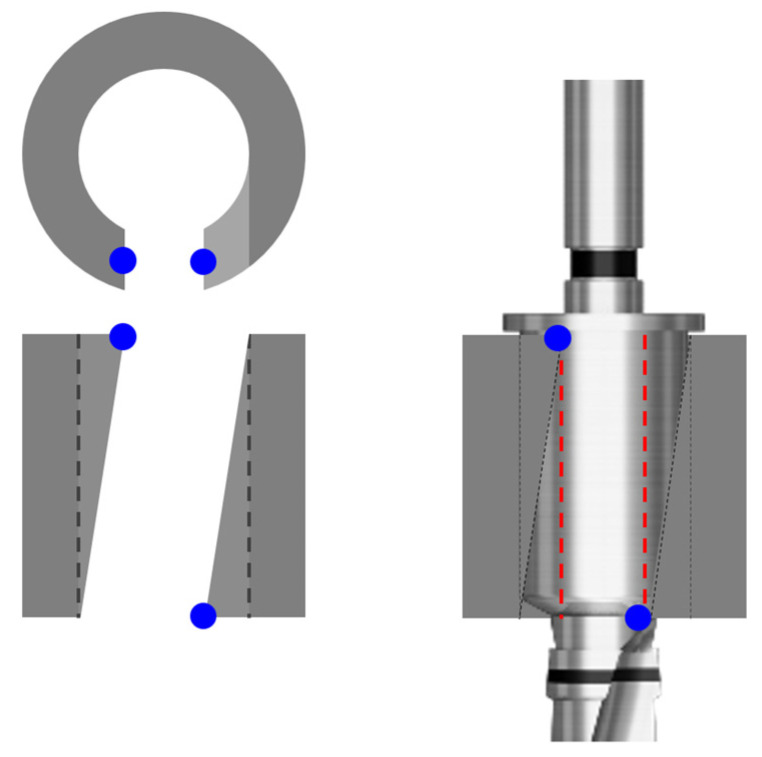
Design of the slope-sleeve. Blue dot indicates barrel holding point.

Furthermore, the slope type exhibited better accuracy compared to the open type, likely due to the intentional design where the direction of the drill entering the guide differed from the actual drilling trajectory. Side openings at the top and bottom of the guide seem to contribute to stabilizing the drill barrel, thereby preventing buccal–lingual deviation (blue dot of Figure 10).

Morón-Conejo et al. [26] have compared DLP type and stereolithographic (SLA) type prints, and they found that the surgical guide printed using a DLP-type printer had a higher accuracy. However, they did not report the accuracy of drilling when the guide was actually used. In our study, experiments were conducted using prints from PolyJet and DLP-type printers. When the overall results were compared with the printer type as the only variable, it was found that using the PolyJet resulted in statistically significantly lower deviations in both horizontal and angle deviations (horizontal deviation: *p* < 0.001; angle deviation: *p* = 0.001). Several studies have compared the accuracy of models obtained through different 3D printing technologies. Ciocan et al. [27] and Wen et al. [28] studied the trueness of models and found that results obtained with the PolyJet printer exhibited better trueness than those obtained with the DLP printer. Similarly, in the study by Graf et al. [29] on dimensional reliability, the PolyJet also showed better results. In light of these findings, the favorable results we obtained with the PolyJet printer can be considered attributable to its higher accuracy compared to the DLP printer.

Findings from this study contribute valuable insights into the performance and limitations of different surgical guide designs, particularly under conditions of limited interocclusal distance. The slope-type guide demonstrated improved accuracy compared to the open type, which could be attributed to its unique design that could better stabilize the drill during the procedure. However, despite these advancements, it is important to recognize the limitations of this study. The accuracy of the surgical guides was assessed in a controlled laboratory environment. Results might vary in clinical settings. Additionally, this study only evaluated the initial drilling procedure. The accuracy may differ during the osteotomy sequence compared to final implant placement. Future research should focus on clinical trials to validate these findings and explore practical implications of these surgical guides in diverse patient populations.

## 5. Conclusions

The findings revealed that the slope-type guide provided superior stability and accuracy to the open type on the drilling process, particularly in challenging clinical scenarios with limited interocclusal distance. Overall, the PolyJet-printed guides displayed enhanced accuracy with the closed type over the open type. For the DLP-printed guides, both the closed and slope types achieved better accuracy than the open type. Across all configurations, PolyJet-printed guides showed statistically lower deviations in horizontal and angular measurements compared to DLP-printed guides, supporting the application of PolyJet technology in high-precision dental procedures. The slope-sleeve surgical guide design, coupled with the appropriate 3D printing technology, can enhance drilling accuracy in the limited interarch space.

## Figures and Tables

**Table 1:** Horizontal deviation of implant drilling with different surgical guides (mm, mean ± SD).

	Closed Type	Slope Type	Open Type	*p*-Value
PolyJet regular	0.37 ± 0.030	0.38 ± 0.053	0.45 ± 0.094	0.65
PolyJet wide	0.28 ± 0.024	0.43 ± 0.070	0.55 ± 0.073	0.015 *
DLP regular	0.59 ± 0.097	0.65 ± 0.082	0.86 ± 0.082	0.84
DLP wide	0.30 ± 0.047	0.34 ± 0.046	0.69 ± 0.093	<0.001 *

SD, standard deviation; DLP, Digital Light Processing. * Significant at *p* < 0.05.

**Table 2:** Angle deviation of implant drilling with different surgical guides (degree, mean ± SD).

	Closed Type	Slope Type	Open Type	*p*-Value
PolyJet regular	1.31 ± 0.105	1.35 ± 0.188	1.59 ± 0.333	0.655
PolyJet wide	1.00 ± 0.086	1.52 ± 0.246	1.95 ± 0.256	0.014 *
DLP regular	2.08 ± 0.346	2.21 ± 0.282	3.06 ± 0.290	0.065
DLP wide	1.08 ± 0.166	1.20 ± 0.163	2.45 ± 0.331	<0.001 *

SD, standard deviation; DLP, Digital Light Processing. * Significant at *p* < 0.05.

**Table 3:** Horizontal and angular deviation of surgical guides fabricated from PolyJet or DLP (mm or degree, mean ± SD).

	PolyJet (*n* = 60)	DLP (*n* = 60)	*p*-Value
Horizontal	0.410 ± 0.206	0.572 ± 0.307	<0.001 *
Angle	1.453 ± 0.727	2.010 ± 1.080	0.001 *

SD, standard deviation; DLP, Digital Light Processing. * Significant at *p* < 0.05.

## Data Availability

The data extracted from the included studies and the data used for the analyses are available upon request from the corresponding authors.
